# Analysis of Radiation Facility Volume and Survival in Men With Lymph Node–Positive Prostate Cancer Treated With Radiation and Androgen Deprivation Therapy

**DOI:** 10.1001/jamanetworkopen.2020.25143

**Published:** 2020-12-10

**Authors:** Sagar A. Patel, Subir Goyal, Yuan Liu, Drew Moghanaki, Pretesh R. Patel, Sheela Hanasoge, Vishal R. Dhere, Jay W. Shelton, Karen D. Godette, Ashesh B. Jani, Bruce Hershatter, Benjamin W. Fischer-Valuck

**Affiliations:** 1 Department of Radiation Oncology, Winship Cancer Institute, Emory University, Atlanta, Georgia; 2 Biostatistics and Bioinformatics Shared Resource, Winship Cancer Institute, Emory University, Atlanta, Georgia; 3 Department of Biostatistics and Bioinformatics, Emory University, Atlanta, Georgia

## Abstract

**Importance:**

Long-term control of node-positive (N1) prostate cancer, the incidence of which is increasing, is obtainable with aggressive treatment, and definitive external beam radiation therapy (EBRT) with long-term androgen deprivation therapy (ADT) is an increasingly preferred option. Caring for these patients is complex and may require resources more readily available at high-volume centers.

**Objective:**

To evaluate the association between radiation facility case volume and overall survival (OS) in men with N1 prostate cancer.

**Design, Setting, and Participants:**

This cohort study included 1899 men diagnosed with T1N1M0 to T4N1M0 prostate cancer treated with curative-intent EBRT and ADT between January 2004 and December 2016 at US facilities reporting to the National Cancer Database. Data analysis was performed from March to June 2020.

**Exposures:**

Treatment at a center with high vs low average cumulative facility volume (ACFV), defined as the total number of prostate radiation cases at an individual patient’s treatment facility from 2004 until the year of that patient’s diagnosis.

**Main Outcomes and Measures:**

OS was assessed between high- vs low-ACFV centers using the Kaplan-Meier method with and without propensity score–based weighted adjustment and multivariable Cox proportional hazards. The nonlinear association between continuous ACFV and OS was examined through a Martingale residual plot, and the optimal ACFV cutoff point that maximized the separation between high vs low ACFV was identified via a bias adjusted log rank test.

**Results:**

A total of 1899 men met inclusion criteria. The median (interquartile range) age was 66 (60-72) years, 1491 (78.5%) were White individuals, and 1145 (60.3%) were treated at nonacademic centers. The optimal ACFV cutoff point was 66.4 patients treated per year. The median OS for patients treated at high-ACFV vs low-ACFV centers was 111.1 (95% CI, 101.5-127.9) months and 92.3 (95% CI, 87.7-103.9) months, respectively (*P* = .01). On multivariable analysis, treatment at a low-ACFV center was associated with increased risk of death (HR, 1.22; 95% CI, 1.02-1.46, *P* = .03) compared with treatment at a high-ACFV center. These results persisted after propensity score–based adjustment.

**Conclusions and Relevance:**

This cohort study found a significant association of facility case volume with long-term outcomes in men with N1 prostate cancer undergoing EBRT with ADT. Specifically, treatment at a facility with high radiation case volume was independently associated with longer OS. Further studies should focus on identifying which factors unique to high-volume centers may be responsible for this benefit.

## Key Points

**Question:**

Is a cancer facility’s radiation case volume associated with long-term outcomes in men with advanced prostate cancer who were treated with primary radiation therapy?

**Findings:**

In this cohort study of 1899 men from a large US cancer database, men with node-positive prostate cancer undergoing curative-intent radiation therapy with concurrent androgen deprivation therapy had significantly improved median survival rates if they were treated at facilities with a high volume of such patients, independent of academic affiliation.

**Meaning:**

Node-positive prostate cancer is a complex disease entity with the potential for long-term disease control with aggressive management, and these findings suggest that treatment at a high-volume radiation center is associated with improved long-term oncologic outcomes.

## Teaser

This cohort study evaluates the association between radiation facility case volume and overall survival among men with node-positive prostate cancer.

## Introduction

Prostate cancer remains the most common malignant neoplasm in men, accounting for 20% of incident cancer cases in male US residents in 2019.^1^ The decline in routine prostate-specific antigen (PSA) screening in the United States since 2012 has altered the landscape of this disease.^2^ Specifically, there has been an increased incidence of advanced prostate cancer, with a concordant decrease in indolent, localized disease.^3,4^ This effect has been more pronounced with the advent of more sensitive advance diagnostic imaging.^5,6^ Although patients with regional (ie, nodal) or distant metastatic disease are considered stage IV by American Joint Committee on Cancer,^7^ many experience long-term survival with aggressive systemic and local management. For nonmetastatic node-positive (N1) prostate cancer in particular, the National Comprehensive Cancer Network revised the historic treatment recommendation of androgen deprivation therapy (ADT) alone to now include combination external beam radiation therapy (EBRT) with concomitant ADT as the preferred treatment option.^8^

Caring for patients with advanced prostate cancer, namely those with N1 prostate cancer who are eligible for curative EBRT with ADT, is complex and requires sophisticated radiation treatment planning and delivery, including dose escalation to radiographically involved lymph nodes as safely deliverable within normal tissue tolerances. Furthermore, the addition of a second-generation anti-androgen, abiraterone, has shown to improve overall survival (OS) in a post hoc analysis in men with N1 prostate cancer.^9^ Although the incidence of N1 prostate cancer was historically low, the rising incidence of these advanced cases in light of reduced PSA screenings as well as the recent guideline recommendation^8^ of definitive EBRT plus ADT as the preferred treatment option highlight the need to optimize management and identify factors associated with long-term outcomes in these patients.

Numerous studies have shown that patients with cancer who are treated at high-volume facilities have higher rates of long-term survival, including those who undergo primary surgery, radiation, or chemotherapy.^10,11,12,13,14,15,16,17,18,19,20^ Whether radiation case volume influences long-term outcomes in men with N1 prostate cancer is unknown. Herein, we examine the association of radiation facility case volume with OS among men with N1 prostate cancer treated with EBRT and ADT. Given the complexity of management of N1 prostate cancer, we hypothesized that men treated at high-volume centers would have improved OS compared with those treated at low-volume centers.

## Methods

### Data Source and Study Population

The National Cancer Database (NCDB), a nationwide hospital-based cancer registry jointly sponsored by the American College of Surgeons and the American Cancer Society, collects data from more than 1400 Commission on Cancer–accredited hospitals and captures approximately 70% of incident cancer cases in the United States. The present study follows the Strengthening the Reporting of Observational Studies in Epidemiology (STROBE) reporting guideline. Because the study used deidentified data from the NDCB database, the requirement for formal institutional review and the need for informed consent were waived, consistent with the policies of Emory University School of Medicine.

Using the NCDB, we identified men diagnosed with T1N1M0 to T4N1M0 prostate adenocarcinoma treated with curative-intent EBRT and concomitant ADT between January 2004 and December 2016. Those who received a total radiation dose of at least 60 Gy were included in the analysis to encompass both moderately hypofractionated and standard fractionated schedules and to exclude incomplete or palliative courses of radiation. Those patients with unknown tumor stage or who underwent surgery, brachytherapy, chemotherapy, or immunotherapy were excluded. Men who initiated ADT more than 1 year before or after the start of radiation were excluded. Patients whose radiation therapy was delivered at multiple places or whose facility information was unknown were also excluded.

### Defining High– vs Low–Treatment Volume Facilities

Before applying exclusion and inclusion criteria, all facilities delivering prostate radiation therapy were included in our initial analysis, and the number of prostate radiation cases for each facility per year was calculated. Given that a facility’s radiation patient volume can vary from year to year, a cumulative facility volume was defined as the total number of radiation cases at an individual patient’s treatment facility from 2004 until the year of that patient’s diagnosis. This cumulative facility volume, specific to each patient, was then divided by the total number of years that the facility reported to the NCDB until that patient’s year of diagnosis. This was subsequently defined as the average cumulative facility volume (ACFV) for that individual patient. Therefore, the ACFV was defined at the level of the patient and represents the experience level of the treating facility at the time that specific patient received treatment. It is therefore possible for patients treated at the same facility to be included as treated at either a high-ACFV center or low-ACFV center based on that individual’s year of diagnosis and that particular facility’s case volume per year leading up to that patient’s treatment. In the final analysis data, the nonlinear association between continuous ACFV and OS was visualized in a Martingale residual plot (eFigure in the Supplement), in which the Martingale residuals were estimated from the Cox proportional hazard model and then plotted against ACFV by local linear regression curve. The optimal ACFV cutoff point that maximizes the separation between the 2 groups (high vs low ACFV) was identified via a bias adjusted log-rank test.^21^ This method enabled the estimation and evaluation of the significance of the cutoff value while controlling for the bias created by the data-driven searching process.

### Statistical Analysis

Descriptive statistics were used to present baseline characteristics. Covariates included facility type (academic vs nonacademic), age at diagnosis, race, primary payer, residential median income, education level, Charlson-Deyo comorbidity score, T stage, Gleason score, PSA level, total radiation dose, year of diagnosis, and distance to treatment center. Analysis of variance and χ^2^ test were used to compare clinical and demographic characteristics between high-volume and low-volume facilities. The primary end point was OS, defined as months from the date of diagnosis to the date of death or last follow-up. Kaplan-Meier curves with and without propensity score–based adjustment using inverse probability score weighting (IPSW) were used to compare OS between those treated at high-volume and low-volume facilities. All measured covariates were used to generate the propensity score. In the propensity score–weighted cohort, the balance of covariates between groups was evaluated by the standardized differences, and a value of less than 0.1 was considered a negligible imbalance. Multivariable Cox proportional hazards, which were built by backward variable selection procedure with an α ≥ .05 removal criteria, were used to compare OS between those who were treated at high-volume vs low-volume facilities. Proportional hazards assumptions were tested using Kolmogorov-type supremum test and were not violated. All analyses were computed using SAS version 9.4 (SAS Institute Inc) from March to June 2020. Tests were 2-sided, with *P* < .05 as the level of significance.

## Results

**Figure 1.**
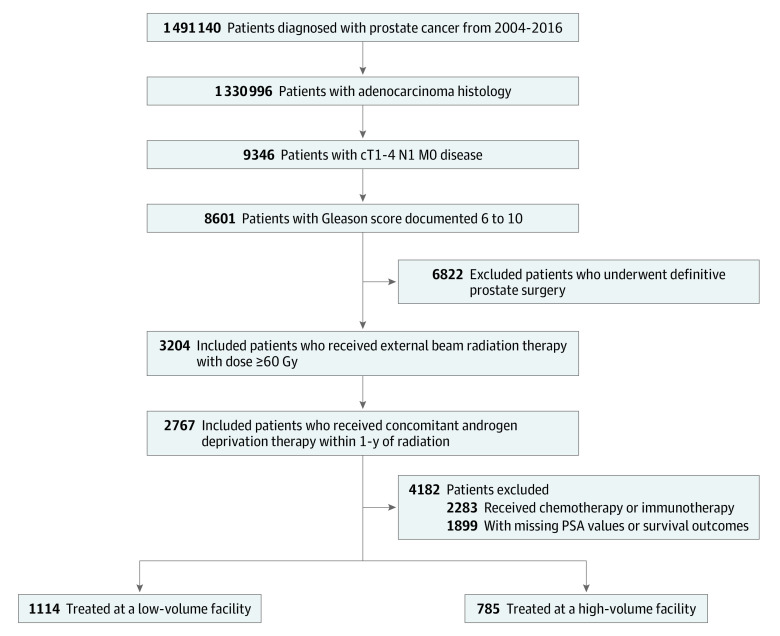
Flow Diagram of Inclusion and Exclusion Criteria PSA indicates prostate-specific antigen.

From the 1 491 140 patients diagnosed with prostate cancer from 2004 to 2016 in the NCDB, we identified 1899 patients who met inclusion criteria (Figure 1). The median (interquartile) age was 66 (60-72) years. Most men were White individuals (1491 [78.5%]) and only 326 (17.2%) were Black individuals. More patients were treated at nonacademic facilities (1145 [60.3%]) than academic or research centers (754 [39.7%]). Baseline clinical and demographic characteristics are shown in the eAppendix in the Supplement. Median follow-up was 102.8 (95% CI, 94.9-109.9) months. The median ACFV was 57.2 cases per year (range, 2-651). The optimal ACFV cutoff point was 66.4 patients treated per year. Overall, 1114 patients (58.7%) were treated at low-ACFV centers, and 785 patients (41.3%) were treated at high-ACFV centers. Patient sociodemographic and clinical characteristics, stratified by ACFV, are summarized in Table 1.

**Table 1.** Univariate Association With Average Cumulative Facility Volume

Characteristic	No. (%), by average cumulative facility volume		Parametric *P* value^a^
	Low (n = 1114)	High (n = 785)	
Facility type			
Nonacademic or research program	775 (69.6)	370 (47.1)	<.001
Academic or research program	339 (30.4)	415 (52.9)	
Age at diagnosis, y			
≤65	561 (50.4)	381 (48.5)	.43
>65	553 (49.6)	404 (51.5)	
Race			
White	876 (78.6)	615 (78.3)	.97
Black	191 (17.1)	135 (17.2)	
Other	47 (4.2)	35 (4.5)	
Primary payer			
Other government, not insured, or unknown	148 (13.3)	65 (8.3)	.001
Private	445 (39.9)	357 (45.5)	
Medicare	521 (46.8)	363 (46.2)	
Median income quartiles, 2008-2012, $			
≥68 000	308 (27.8)	333 (42.9)	<.001
48 000-67 999	312 (28.2)	195 (25.1)	
38 000-47 999	298 (26.9)	138 (17.8)	
<38 000	190 (17.1)	111 (14.3)	
Residents without high school degree, 2008-2012, %			
<7.0	267 (24.1)	234 (30.0)	.004
7.0-12.9	366 (33)	259 (33.2)	
13.0-20.9	275 (24.8)	183 (23.5)	
≥21	201 (18.1)	103 (13.2)	
Charlson-Deyo score			
0	935 (83.9)	656 (83.6)	.38
1	140 (12.6)	109 (13.9)	
≥2	39 (3.5)	20 (2.5)	
AJCC clinical T stage			
1	257 (23.1)	169 (21.5)	.39
2	367 (32.9)	246 (31.3)	
3-4	490 (44)	370 (47.1)	
Gleason score			
6-7	282 (25.3)	151 (19.2)	.002
8-10	832 (74.7)	634 (80.8)	
PSA level, ng/mL			
<10	307 (27.6)	242 (30.8)	.26
10-20	268 (24.1)	188 (23.9)	
>20	539 (48.4)	355 (45.2)	
Regional with boost radiation dose, Gy			
Median (IQR) [minimum]	77.4 (75.6-79.2) [60.0]	77.4 (75.6-79.2) [60.0]	.58
Total radiation dose, Gy			
<74	228 (20.5)	125 (15.9)	.01
≥74	886 (79.5)	660 (84.1)	
Year of diagnosis			
2004-2008	303 (27.2)	219 (27.9)	.72
2009-2011	270 (24.2)	192 (24.5)	
2012-2014	362 (32.5)	263 (33.5)	
2015	179 (16.1)	111 (14.1)	
Great circle distance, mi			
0.2-4.9	300 (27.1)	184 (23.5)	.046
5.0-10.5	276 (24.9)	191 (24.4)	
10.6-24.3	281 (25.3)	187 (23.9)	
24.4-1856.8	252 (22.7)	220 (28.1)	

Abbreviations: AJCC, American Joint Committee on Cancer; IQR, interquartile range; PSA, prostate-specific antigen level.

SI conversion factor: To convert PSA level to micrograms per liter, multiply by 1.0.

^a^ The parametric *P* value is calculated by analysis of variance for numerical covariates and the χ^2^ test for categorical covariates.

Compared with low-ACFV centers, high-ACFV centers were more likely to be academic or research programs (339 [30.4%] vs 415 [52.9%]; *P* < .001). High-ACFV sites more commonly treated patients with private insurance than low-ACFV sites (357 [45.5%] vs 445 [39.9%]; *P* = .001), and less commonly treated patients without insurance, another type of government insurance, or unknown insurance (65 [8.3%] vs 148 [13.3%]; *P* = .001). Patients treated at high-ACFV centers had higher median incomes than those treated at low-ACFV centers (≥$68 000 median income, 333 [42.9%] vs 308 [27.8%]; *P* < .001) and higher education level as measured by percentage of patients without high school degree (<7% without high school degree, 234 [30.0%] vs 267 [24.1%]; *P* = .004). High-ACFV centers also treated a higher proportion of patients with Gleason scores between 8 and 10 than low-ACFV centers (634 [80.8%] vs 832 [74.7%]; *P* = .002) and more commonly delivered total radiation doses of at least 74 Gy (660 [84.1%] vs 886 [79.5%]; *P* = .01). Patients whose home residence zip code was more than 24.4 miles from their treatment facility were more commonly treated at high-ACFV centers than low-ACFV centers (220 [28.1%] vs 252 [22.7%]; *P* = .046). Age, race, Charlson-Deyo Score, year of diagnosis, and PSA did not differ between patients treated at high- and low-ACFV facilities. There was no difference between time from diagnosis to initiating ADT between low- and high-ACFV facilities (median [IQR], 35 [19-59] days vs 36 [21-60] days) or time from diagnosis to starting radiation therapy (median [IQR], 118 [88-159] days vs 118 [90-157] days). After IPSW adjustment, all baseline characteristics were distributed evenly, with an absolute standard difference of less than 0.1 between the high- and low-ACFV centers (eAppendix in the Supplement).

**Figure 2.**
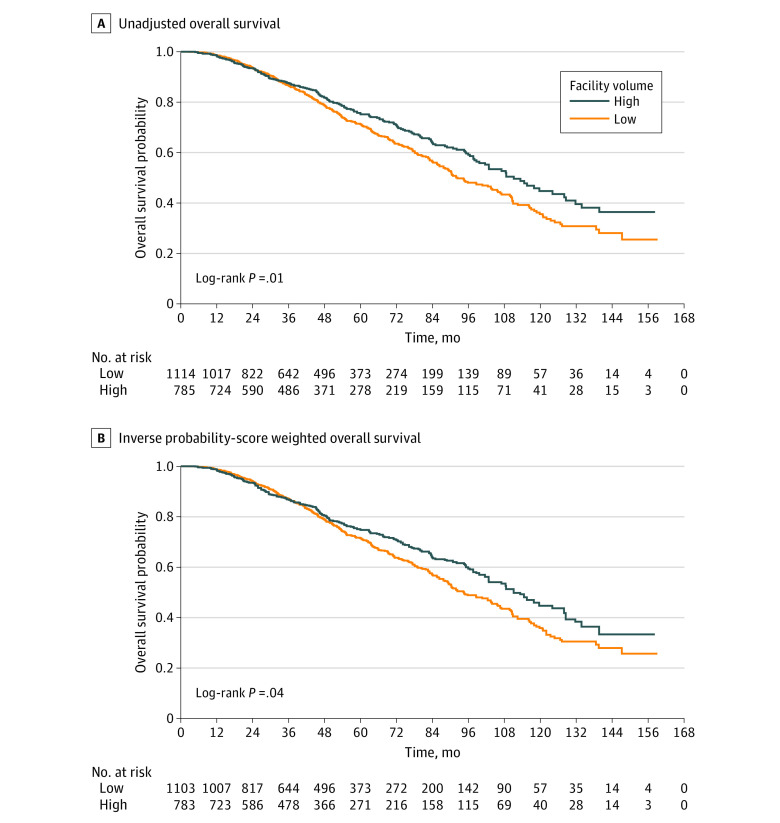
Overall Survival

The median OS for patients treated at high-ACFV vs low-ACFV centers was 111.1 (95% CI, 101.5-127.9) months vs 92.3 (95% CI, 87.7-103.9) months (*P* = .01) (Figure 2A). The estimated 10-year OS rate for patients treated at high-ACFV vs low-ACFV centers was 44.7% (95% CI, 37.7%-51.6%) vs 35.6% (95% CI, 30.1%-41.1%). This OS benefit persisted after IPSW adjustment, with a weighted median OS for patients treated at high-ACFV centers of 111.1 (95% CI, 101.5-127.9) months compared with 94.5 (95% CI, 88.2-105.8) months for patients treated at low-ACFV centers (*P* = .04) (Figure 2B).

On multivariable Cox regression, treatment at a low-ACFV center was associated with increased risk of death (hazard ratio [HR], 1.22; 95% CI, 1.02-1.46; *P* = .03) compared with treatment at a high-ACFV center. Other covariates on multivariable analysis that were associated with a lower overall survival rate included Medicare insurance, treatment at nonacademic or research programs, increasing comorbidity score, increasing clinical T stage, and Gleason score 8 to 10 (Table 2). On IPSW analysis, a lower OS rate was again observed in patients treated at low-ACFV centers compared with high-ACFV centers (adjusted HR [aHR], 1.20; 95% CI, 1.01-1.43; *P* = .04). In the weighted analysis, treatment at a nonacademic or research facility and Medicare insurance status were no longer associated with lower survival. Increasing age was associated with lower OS (aged ≤65 years old: aHR, 0.69; 95% CI, 0.58-0.82; *P* < .001). Increasing comorbidity score, increasing clinical T stage, and Gleason score 8 to 10 were also associated with lower OS in the IPSW model (Table 3). PSA value (ie, <10, 10-20, or >20 ng/mL [to convert to micrograms per liter, multiply by 1.0]) and total radiation dose delivered (<74 Gy or ≥74 Gy) were not statistically significantly associated with survival in either unweighted or weighted analyses.

**Table 2.** Multivariable Analysis Cox Proportional Hazard Model for OS^a^

Characteristic	OS		
	HR (95% CI)	*P* value	
		HR	Overall
Average cumulative facility volume			
Low	1.22 (1.02-1.46)	.03	.03
High	1 [Reference]	NA	
Primary payer			
Other government, not insured, or unknown	0.62 (0.45-0.85)	.003	<.001
Private	0.67 (0.56-0.80)	<.001	
Medicare	1 [Reference]	NA	
Facility type			
Nonacademic or research program	1.23 (1.02-1.48)	.03	.03
Academic or research program	1 [Reference]	NA	
Charlson-Deyo Score			
0	0.45 (0.30-0.68)	<.001	<.001
1	0.62 (0.39-0.97)	.04	
≥2	1 [Reference]	NA	
AJCC clinical T stage			
1	0.73 (0.58-0.92)	.007	.001
2	0.73 (0.61-0.89)	.002	
3-4	1 [Reference]	NA	
Gleason score			
6-7	0.67 (0.54-0.83)	<.001	<.001
8-10	1 [Reference]	NA	

Abbreviations: AJCC, American Joint Committee on Cancer; HR, hazard ratio; NA, not applicable; OS, overall survival.

^a^ A total of 1899 observations were in the original data set and used in this analysis. Backward selection with an α < .05 was used. The following variables were removed from the model: age at diagnosis, median income quartiles, residents with no high school degree, prostate-specific antigen levels, race, and total radiation dose (ie, <74 vs ≥74 Gy).

**Table 3.** Multivariable Analysis With OS in Weighted Sample^a^

Characteristic	OS		
	HR (95% CI)	*P* value	
		HR	Overall
Average cumulative facility volume			
Low	1.20 (1.01-1.43)	.04	.04
High	1 [Reference]	NA	
Age at diagnosis, y			
≤65	0.69 (0.58-0.82)	<.001	<.001
>65	1 [Reference]	NA	
Median income quartiles, 2008-2012, $			
≥68 000	1.02 (0.78-1.34)	.86	.02
48 000-67 999	1.18 (0.90-1.55)	.23	
38 000-47 999	1.42 (1.08-1.86)	.01	
<38 000	1 [Reference]	NA	
Charlson-Deyo score			
0	0.41 (0.28-0.62)	<.001	<.001
1	0.58 (0.37-0.91)	.02	
≥2	1 [Reference]	NA	
AJCC clinical T stage			
1	0.77 (0.62-0.98)	.03	.01
2	0.76 (0.63-0.92)	.006	
3-4	1 [Reference]	NA	
Gleason score			
6-7	0.67 (0.55-0.83)	<.001	<.001
8-10	1 [Reference]	NA	

Abbreviations: AJCC, American Joint Committee on Cancer; HR, hazard ratio; NA, not applicable; OS, overall survival.

^a^ A total of 1885 were in the data set and used in this analysis Backward selection with an α < .05 was used. The following variables were removed from the model: great circle distance, facility type, primary payer, residents with no high school degree, prostate-specific antigen level, race, year of diagnosis, and total radiation dose (ie, <74 vs ≥74 Gy).

### Discussion

The management of N1 prostate cancer has historically been controversial, but despite lack of randomized trials investigating the role of aggressive local treatment, recent guidelines^8^ have included the addition of pelvic EBRT with long-term ADT as the preferred treatment option. Several retrospective analyses have shown a significantly higher rate of failure-free survival or OS in patients who received EBRT in addition to ADT that support the recent inclusion of EBRT in the treatment algorithm.^22,23,24,25^ For example, post hoc analysis of the STAMPEDE trial^22^ showed that the addition of EBRT in patients with N1 prostate cancer resulted in an approximate 50% reduction in risk of failure at a median 1.5-year follow-up compared with systemic therapy alone. Population-based analyses using SEER and NCDB^23,24,25^ have shown higher rates of both prostate-specific survival and OS with the addition of EBRT to ADT. Optimizing radiation management and identifying factors associated with long-term outcomes is paramount with this evolving treatment strategy.

Our analysis showed a significant association of facility volume with long-term outcomes in men with N1 prostate cancer undergoing EBRT with concomitant ADT. Specifically, treatment at a low-volume facility was associated with a 20% lower OS rate compared with treatment at a high-volume facility. Our findings are similar to those from numerous studies establishing an association between hospital and/or surgeon volume and survival for various cancer types.^10,11,12,13,14,15,16^ To our knowledge, our study is among few large population-based analyses to show an association between case volume and survival in patients receiving radiation. Similar to our cohort of patients with N1 prostate cancer, this association has been demonstrated in other aggressive disease types, including locally advanced lung and head and neck cancers, muscle invasive bladder cancer, and high-risk prostate cancer.^17,18,19,20^

There are several possible explanations for this association. First, N1 prostate cancer, as with other advanced cancers eligible for definitive therapy, requires complex management decisions, including advanced treatment planning and delivery. In light of the radiobiologic properties of prostate cancer, dose escalation to the prostate gland has been well established to improve long-term disease control.^26,27,28^ Standard elective radiation doses to metastatically involved lymph nodes may not be sufficiently cytotoxic, and dose escalation to gross disease in pelvic lymph nodes may be considered. In fact, 2019 evidence-based guidelines from Australia and New Zealand now advocate for advanced molecular imaging for men with N1 prostate cancer and subsequently treating elective pelvic lymph node basins with dose escalation to positron emission tomography (PET)–avid lymph nodes in addition to the prostate.^29^

Nodal dose escalation is highly variable in the absence of defined clinical trial protocols, and typically it is acceptable to treat patients with as high as dose-volume histogram parameters and normal tissue tolerance (eg, bowel, bladder, and rectum) allow. With advanced treatment delivery platforms, such as volumetric arc therapy (VMAT) with image-guidance (IGRT), in conjunction with physics and dosimetry expertise, this is often accomplished via a simultaneous integrated boost to involved lymph nodes while delivering lower doses to uninvolved obturator and the internal/external iliac chain. The technique and dose is ultimately left to the discretion of the treating radiation oncologist; it is plausible that physicians at high-volume centers with VMAT and IGRT capabilities may feel more comfortable delivering higher doses to targets (ie, involved lymph nodes, prostate, seminal vesicles) despite the higher risk of complications associated with it. While the details of treatment techniques are unavailable in the NCDB, a greater percentage of men received a 74 Gy or greater boost at high-volume than low-volume centers. The NCDB does not provide details on whether the cumulative boost doses was to the prostate with or without radiographic lymph nodes; however, it is possible that dose escalation to all sites of gross disease is beneficial for long-term cancer control.

There are other aspects of care at high-volume centers, outside of radiation treatment and not quantifiable in the NCDB, that may explain the higher long-term survival rate seen in men with N1 prostate cancer. For one, high-volume facilities may harbor optimal multidisciplinary care in the same hospital and center. Treatment of patients with advanced prostate cancer requires close collaboration between urologists, radiation oncologists, medical oncologists, radiologists, and pathologists.^30^ Each member of this complex team contributes to timely diagnosis, optimal staging, early initiation of therapy, and posttreatment surveillance. It is plausible that high-volume centers more often have closer collaboration and workflows between these disciplines, including the establishment of multidisciplinary clinics and tumor boards. Second, high-volume centers may more routinely use advanced molecular imaging, such as fluciclovine, choline, or prostate-specific membrane antigen PET, that may detect occult nodal disease, resulting in stage migration and overall better outcomes compared with men who have more advanced nodal disease burden that is detectable by conventional imaging. Additionally, advanced PET imaging can be used during radiation treatment planning to deliver higher doses to sites of tracer-avid lymph nodes, which may otherwise go undetected with standard pelvic computed tomography–guided treatment. Third, medical oncologists at high-volume centers may have more expertise with authorizing and using advanced systemic agents, such as abiraterone, which have been demonstrated to improve survival in N1 prostate cancer, not to mention several others that are now approved in the recurrent and metastatic setting.^9^ Fourth, clinical staff, including advanced practitioners and nurses, at high-volume centers may have more experience with managing acute toxic effects associated with aggressive local and systemic therapy, and adherence to treatment completion may subsequently be higher. It is important to note that many of these characteristics are also representative of academic centers with specialized care; however, we observed the association of case volume with long-term survival even after adjusting for academic vs nonacademic facilities.

### Limitations

This study has several limitations. First, the NCDB is a hospital-based cancer registry that captures only patients who are diagnosed or treated at Commission on Cancer–accredited facilities. These results may not represent the entire cancer population in the United States; however, given that the NCDB includes approximately 70% of all newly diagnosed cancer cases each year, we believe that this analysis is a notable reflection of outcomes between other high-volume and low-volume facilities in the United States that may not be captured in the registry. Second, outcome measures in the NCDB are limited to OS, so details regarding biochemical control or cancer-specific survival are unavailable. However, we believe OS is the criterion standard end point in men with N1 prostate cancer given the advanced nature of the disease with a high likelihood of distant metastatic progression and death. Third, toxic effects and quality-of-life measurements are unavailable in the NCDB and could not be assessed; it is plausible that the improved survival at high-volume centers is associated with more aggressive therapy and subsequent worse acute toxic effects and quality of life. Fourth, some important details regarding systemic therapy are lacking in the NCDB. Specifically, duration of ADT, which could affect long-term outcomes, is unavailable and could not be accounted for. Additionally, details regarding chemotherapy use, including type of agent and number of cycles, are unavailable; therefore, we excluded patients who received any chemotherapy to avoid additional unmeasured treatment confounders, given that different chemotherapy drugs can have variable cytotoxic effects in prostate cancer. But chemotherapy may be associated with OS in this population, and studies that can include specific details regarding the chemotherapy regimen provided to men with N1 prostate cancer are warranted. Fifth, given the retrospective design using a population-based database, analyses are subject to selection biases and imbalances in unmeasured variables. While multivariate modeling and propensity score matching including all available clinical factors associated with prostate cancer outcomes were accounted for, some data unavailable in the NCDB, such as radiographic size or number of involved lymph nodes, were unable to be accounted for and could affect outcomes.

## Conclusions

N1 prostate cancer is a complex disease entity with the potential for long-term disease control with aggressive management. This study found that for men with N1 prostate cancer, treatment at a facility with high radiation case volume, independent of academic affiliation, was associated with longer OS. Considering that definitive EBRT with ADT is an increasingly preferred treatment option for these men, our results are hypothesis-generating, and further studies should focus on identifying which factors unique to high-volume centers may be responsible for this benefit.

## Notes

Supplement. **eAppendix.** Overall Sample Distribution and Balance Check for Before and After IPSW **eFigure. **Martingale Residual Plot

## References

[1] Siegel RL, Miller KD, Jemal A Cancer statistics, 2019. CA Cancer J Clin. 2019;69(1):7-34. doi:10.3322/caac.21551 30620402

[2] Jemal A, Fedewa SA, Ma J, Prostate cancer incidence and PSA testing patterns in relation to USPSTF screening recommendations. JAMA. 2015;314(19):2054-2061. doi:10.1001/jama.2015.14905 26575061

[3] Butler SS, Muralidhar V, Zhao SG, Prostate cancer incidence across stage, NCCN risk groups, and age before and after USPSTF Grade D recommendations against prostate-specific antigen screening in 2012. Cancer. 2020;126(4):717-724. doi:10.1002/cncr.32604 31794057

[4] Houston KA, King J, Li J, Jemal A Trends in prostate cancer incidence rates and prevalence of prostate specific antigen screening by socioeconomic status and regions in the United States, 2004 to 2013. J Urol. 2018;199(3):676-682. doi:10.1016/j.juro.2017.09.103 28965781 PMC11181155

[5] Li R, Ravizzini GC, Gorin MA, The use of PET/CT in prostate cancer. Prostate Cancer Prostatic Dis. 2018;21(1):4-21. 29230009 10.1038/s41391-017-0007-8

[6] Alemozaffar M, Akintayo AA, Abiodun-Ojo OA, [18F]Fluciclovine positron emission tomography/computerized tomography for preoperative staging in patient with intermediate to high risk primary prostate cancer. J Urol. 2020;204(4):737-740. doi:10.1097/JU.0000000000001095 32347780 PMC8059474

[7] Buyyounouski MK, Choyke PL, McKenney JK, Prostate cancer—major changes in the American Joint Committee on Cancer eighth edition cancer staging manual. CA Cancer J Clin. 2017;67(3):245-253. doi:10.3322/caac.21391 28222223 PMC6375094

[8] National Comprehensive Cancer Network Prostate Cancer (NCCN Guidelines Version 3.2020). Accessed November 9, 2020 https://www.nccn.org/professionals/physician_gls/pdf/prostate.pdf

[9] James ND, de Bono JS, Spears MR, ; STAMPEDE Investigators Abiraterone for prostate cancer not previously treated with hormone therapy. N Engl J Med. 2017;377(4):338-351. doi:10.1056/NEJMoa1702900 28578639 PMC5533216

[10] Liu CJ, Chou YJ, Teng CJ, Association of surgeon volume and hospital volume with the outcome of patients receiving definitive surgery for colorectal cancer: a nationwide population-based study. Cancer. 2015;121(16):2782-2790. doi:10.1002/cncr.29356 25892632

[11] Lüchtenborg M, Riaz SP, Coupland VH, High procedure volume is strongly associated with improved survival after lung cancer surgery. J Clin Oncol. 2013;31(25):3141-3146. doi:10.1200/JCO.2013.49.0219 23897962

[12] Schrag D, Cramer LD, Bach PB, Cohen AM, Warren JL, Begg CB Influence of hospital procedure volume on outcomes following surgery for colon cancer. JAMA. 2000;284(23):3028-3035. doi:10.1001/jama.284.23.3028 11122590

[13] Bach PB, Cramer LD, Schrag D, Downey RJ, Gelfand SE, Begg CB The influence of hospital volume on survival after resection for lung cancer. N Engl J Med. 2001;345(3):181-188. doi:10.1056/NEJM200107193450306 11463014

[14] Ellison LM, Heaney JA, Birkmeyer JD The effect of hospital volume on mortality and resource use after radical prostatectomy. J Urol. 2000;163(3):867-869. 10687994

[15] Go RS, Bartley AC, Crowson CS, Association between treatment facility volume and mortality of patients with multiple myeloma. J Clin Oncol. 2017;35(6):598-604. doi:10.1200/JCO.2016.68.3805 28199819

[16] Liu T, David M, Ellis O, Treatment for oral squamous cell carcinoma: Impact of surgeon volume on survival. Oral Oncol. 2019;96:60-65. doi:10.1016/j.oraloncology.2019.06.030 31422214

[17] David JM, Ho AS, Luu M, Treatment at high-volume facilities and academic centers is independently associated with improved survival in patients with locally advanced head and neck cancer. Cancer. 2017;123(20):3933-3942. doi:10.1002/cncr.30843 28640546

[18] Wang EH, Rutter CE, Corso CD, Patients selected for definitive concurrent chemoradiation at high-volume facilities achieve improved survival in stage III non-small-cell lung cancer. J Thorac Oncol. 2015;10(6):937-943. doi:10.1097/JTO.0000000000000519 25738221

[19] Chen YW, Mahal BA, Muralidhar V, Association between treatment at a high-volume facility and improved survival for radiation-treated men with high-risk prostate cancer. Int J Radiat Oncol Biol Phys. 2016;94(4):683-690. doi:10.1016/j.ijrobp.2015.12.008 26972640

[20] D’Rummo KA, TenNapel MJ, Shen X The impact of radiotherapy facility volume on the survival and guideline concordance of patients with muscle-invasive bladder cancer receiving bladder-preservation therapy. Am J Clin Oncol. 2019;42(9):705-710. doi:10.1097/COC.0000000000000582 31368905

[21] Contal C, O’Quigley J An application of changepoint methods in studying the effect of age on survival in breast cancer. Comput Stat Data Anal. 1999;30(3):253-270. doi:10.1016/S0167-9473(98)00096-6

[22] James ND, Spears MR, Clarke NW, ; STAMPEDE Investigators Failure-free survival and radiotherapy in patients with newly diagnosed nonmetastatic prostate cancer: data from patients in the control arm of the STAMPEDE Trial. JAMA Oncol. 2016;2(3):348-357. doi:10.1001/jamaoncol.2015.4350 26606329 PMC4789485

[23] Rusthoven CG, Carlson JA, Waxweiler TV, The impact of definitive local therapy for lymph node-positive prostate cancer: a population-based study. Int J Radiat Oncol Biol Phys. 2014;88(5):1064-1073. doi:10.1016/j.ijrobp.2014.01.008 24661660

[24] Tward JD, Kokeny KE, Shrieve DC Radiation therapy for clinically node-positive prostate adenocarcinoma is correlated with improved overall and prostate cancer-specific survival. Pract Radiat Oncol. 2013;3(3):234-240. doi:10.1016/j.prro.2012.11.011 24674370

[25] Lin CC, Gray PJ, Jemal A, Efstathiou JA Androgen deprivation with or without radiation therapy for clinically node-positive prostate cancer. J Natl Cancer Inst. 2015;107(7):djv119. doi:10.1093/jnci/djv119 25957435

[26] Michalski JM, Moughan J, Purdy J, Effect of standard vs dose-escalated radiation therapy for patients with intermediate-risk prostate cancer: the NRG Oncology RTOG 0126 randomized clinical trial. JAMA Oncol. 2018;4(6):e180039. doi:10.1001/jamaoncol.2018.0039 29543933 PMC5885160

[27] Kuban DA, Tucker SL, Dong L, Long-term results of the M. D. Anderson randomized dose-escalation trial for prostate cancer. Int J Radiat Oncol Biol Phys. 2008;70(1):67-74. doi:10.1016/j.ijrobp.2007.06.054 17765406

[28] Zietman AL, Bae K, Slater JD, Randomized trial comparing conventional-dose with high-dose conformal radiation therapy in early-stage adenocarcinoma of the prostate: long-term results from Proton Radiation Oncology Group/American College of Radiology 95-09. J Clin Oncol. 2010;28(7):1106-1111. doi:10.1200/JCO.2009.25.8475 20124169 PMC2834463

[29] Lieng H, Kneebone A, Hayden AJ, Radiotherapy for node-positive prostate cancer: 2019 recommendations of the Australian and New Zealand Radiation Oncology Genito-Urinary group. Radiother Oncol. 2019;140:68-75. doi:10.1016/j.radonc.2019.05.016 31177044

[30] Gomella LG, Lin J, Hoffman-Censits J, Enhancing prostate cancer care through the multidisciplinary clinic approach: a 15-year experience. J Oncol Pract. 2010;6(6):e5-e10. doi:10.1200/JOP.2010.000071 21358951 PMC2988679

