# Supplementary material for: Analysis of Radiation Facility Volume and Survival in Men With Lymph Node–Positive Prostate Cancer Treated With Radiation and Androgen Deprivation Therapy
Source: JAMA Netw Open. 2020 Dec 10;3(12):e2025143. doi: 10.1001/jamanetworkopen.2020.25143 (PMC7729429; doi:10.1001/jamanetworkopen.2020.25143)
Supplement: Supplement. — eAppendix. Overall Sample Distribution and Balance Check for Before and After IPSW eFigure. Martingale Residual Plot [file jamanetwopen-e2025143-s001.pdf]

Supplemental Online Content

Patel SA, Goyal S, Liu Y, et al. Analysis of radiation facility volume and survival in men with lymph node–positive prostate cancer treated with radiation and androgen deprivation therapy. *JAMA Netw Open*. 2020;3(12):e2025143. doi:10.1001/jamanetworkopen.2020.25143

eAppendix. Overall Sample Distribution and Balance Check for Before and After IPSW
eFigure. Martingale Residual Plot

This supplemental material has been provided by the authors to give readers additional information about their work.

© 2020 Patel SA et al. *JAMA Network Open.*

eAppendix. Overall Sample Distribution and Balance Check for Before and After IPSW

		Study Sample Distribution		Absolute Standardized Difference(ASD)	
**Covariate**	**Level**	**Before**	**After**	**Before**	**After**
Average Cumulative Facility Volume	Overall	1885 (100.0)	1886 (100.0)	-	-
	Low	1108 (58.8)	1103 (58.5)	-	-
	High	777 (41.2)	783 (41.5)	-	-
Age at Diagnosis (years)	≤65	933 (49.5)	937 (49.7)	0.038	0.007
	>65	952 (50.5)	949 (50.3)	0.038	0.007
Race	White	1480 (78.5)	1471 (78)	0.006	0.012
	Black	323 (17.1)	330 (17.5)	0.001	0.01
	Other	82 (4.4)	84 (4.4)	0.013	0.006
Median Income Quartiles 2008-2012	≥$68,000	641 (34)	640 (33.9)	**0.319**	0.004
	$48,000-$67,999	507 (26.9)	499 (26.5)	0.069	0.009
	$38,000-$47,999	436 (23.1)	441 (23.4)	**0.221**	0.002
	<$38,000	301 (16)	306 (16.2)	0.079	0.008
Percent No High School Degree 2008-2012	<7.0%	498 (26.4)	503 (26.7)	**0.132**	0.005
	7.0-12.9%	625 (33.2)	611 (32.4)	0.006	0.01
	13.0-20.9%	458 (24.3)	462 (24.5)	0.03	0.004
	≥=21%	304 (16.1)	309 (16.4)	**0.134**	0.002
Primary Payor	Other Government/Not Insured/Unk	210 (11.1)	217 (11.5)	**0.153**	0.015
	Private	795 (42.2)	794 (42.1)	**0.108**	0.001
	Medicare	880 (46.7)	875 (46.4)	0.012	0.009
Facility Type	Non-Academic/Research Program	1135 (60.2)	1148 (60.9)	**0.474**	0.007
	Academic/Research Program	750 (39.8)	738 (39.1)	**0.474**	0.007
Charlson-Deyo Score	0	1579 (83.8)	1586 (84.1)	0.011	0.016
	1	248 (13.2)	244 (13)	0.037	0.013
	2+	58 (3.1)	55 (2.9)	0.05	0.008

© 2020 Patel SA et al. *JAMA Network Open.*

AJCC Clinical T	T1	425 (22.5)	420 (22.3)	0.038	0.011
	T2	608 (32.3)	610 (32.4)	0.036	0.002
	T3-4	852 (45.2)	856 (45.4)	0.065	0.007
PSA	<10	543 (28.8)	531 (28.2)	0.073	0
	10-20	455 (24.1)	452 (23.9)	0.003	0.004
	>20	887 (47.1)	903 (47.9)	0.064	0.003
Gleason score	6-7	432 (22.9)	432 (22.9)	**0.143**	0
	8-10	1453 (77.1)	1453 (77.1)	**0.143**	0
Total Radiation Dose (cat.)	<74	350 (18.6)	354 (18.8)	**0.115**	0.001
	≥74	1535 (81.4)	1532 (81.2)	**0.115**	0.001
Year of Diagnosis (quartile)	2004 - 2008	514 (27.3)	514 (27.2)	0.01	0.005
	2009 - 2011	458 (24.3)	457 (24.2)	0.006	0.005
	2012 - 2014	623 (33.1)	630 (33.4)	0.024	0.005
	2015	290 (15.4)	286 (15.2)	0.052	0.006
Great Circle Distance (quartile)	0.2 - 4.9	484 (25.7)	488 (25.9)	0.078	0.006
	5.0 - 10.5	465 (24.7)	475 (25.2)	0.009	0.002
	10.6 - 24.3	468 (24.8)	472 (25.1)	0.03	0.004
	24.4 - 1856.8	468 (24.8)	450 (23.9)	**0.117**	0.001
*The absolute standardized Difference (ASD) >= 0.1 is bold and indicates insufficient balance.					

© 2020 Patel SA et al. *JAMA Network Open.*

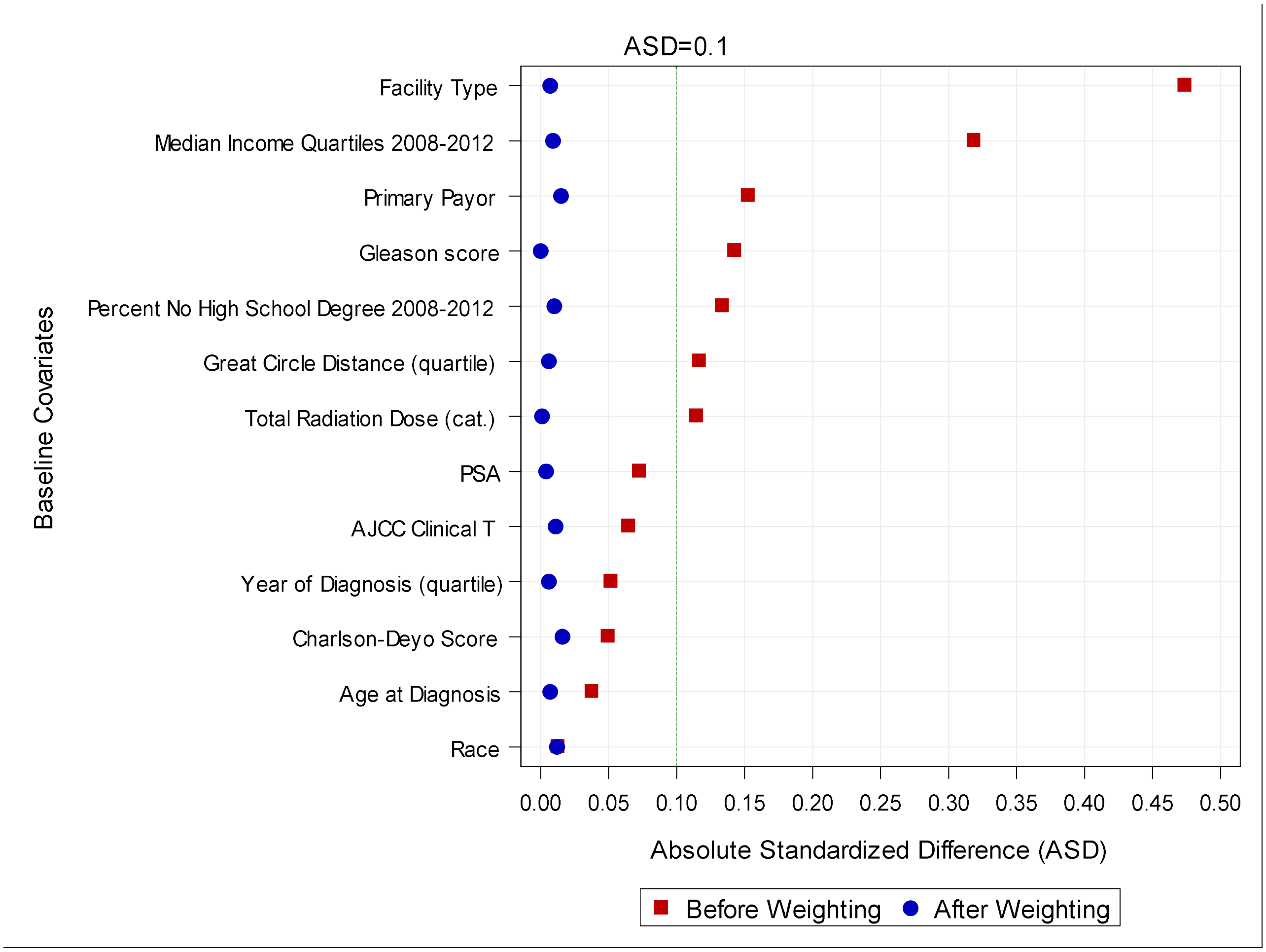

© 2020 Patel SA et al. *JAMA Network Open.*

eFigure. Martingale Residual Plot

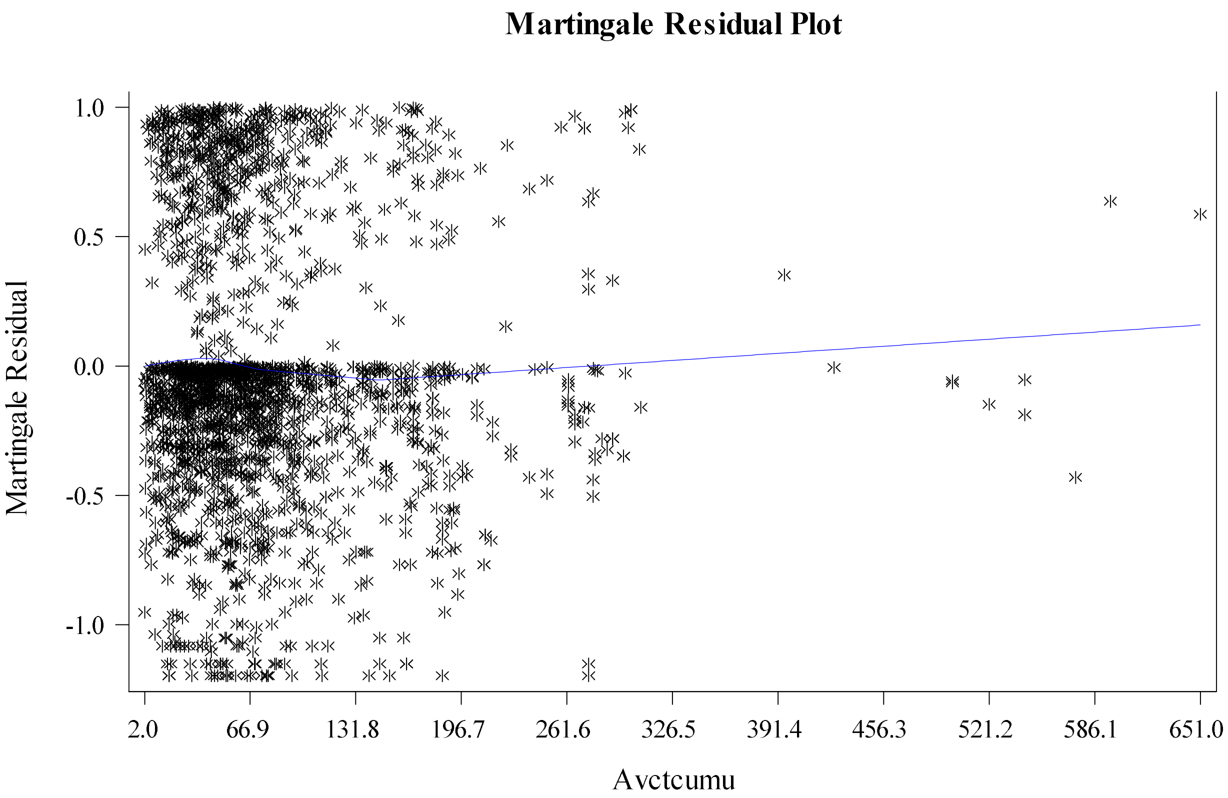

Percentiles

N	Min	10th	20th	30th	40th	50th	60th	70th	80th	90th	Max
1899	2	19.3333	29	39	47.6667	57.2222	68.25	81.3636	105.125	162.25	651

© 2020 Patel SA et al. *JAMA Network Open.*
